# Enhancing the Forming Accuracy of CFRP through a Global Compensation Method by Introducing an Optimal Profile

**DOI:** 10.3390/polym16131792

**Published:** 2024-06-25

**Authors:** Yongming Zhang, Luling An, Cong Zhao

**Affiliations:** Collage of Mechanical & Electrical Engineering, Nanjing University of Aeronautics and Astronautics, Nanjing 210016, China; zymging@163.com (Y.Z.); zhaocong_ccme@nuaa.edu.cn (C.Z.)

**Keywords:** composite structures, curing distortion, coordinated model, global compensation, finite element analysis

## Abstract

Irreversible curing distortion represents a significant limiting factor in the application of high-performance composite structures. Curing distortion is the deviation of a component’s profile from the theoretical profile after demolding. Introducing the optimal compensation profile into the traditional compensation algorithm represents an effective method to enhance CFRPs’ forming accuracy. For this method, it is necessary to obtain the optimal compensating profile by establishing the coordinate model of the curing process parameter and mold profile compensation. The coordinated control model consists of four parameters: the mean value (D_av_), root mean square value (D_msr_), minimum (D_min_), and maximum (D_max_) of curing distortion. Two sizes of composite structural parts are manufactured using the global compensation method. We investigate the influence mechanisms of heating, holding, and cooling times on curing distortion and residual stresses and develop a multi-field coupled finite element model. Strong agreement between the numerical and experimental findings serves as evidence for the effectiveness of the numerical model. The middle layer of the fabricated parts exhibit a reduction in residual stresses as the heating and holding times increase, while an opposite trend is noted with an increase in cooling time. Refining the design of curing process parameters can yield the minimum value of curing deformation within the specified resin system interval. Comparisons indicate that the distortion of the composite wall panel structure is reduced by 86.2% through the use of the global compensation method, demonstrating the validity of this approach for composite structures.

## 1. Introduction

Continuous carbon fiber reinforced polymer (CFRP) composite materials exhibit excellent specific strength and stiffness properties [1,2]. Nowadays, composite materials are extensively employed in various spacecraft, including the A380. In the aerospace industry, wall panel structures are widely used as essential load-bearing components. Utilizing composite materials for the construction of these panels has led to significant weight reduction [3]. The flight efficiency of the aircraft has increased. Composite wall panel structures are generally made up of variable thicknesses of skins, stiffeners, honeycomb or foam filling materials, etc., have complex cross-sections, and are large-scale. Autoclave process technology is a commonly used method for manufacturing composite materials. During the composite curing process in an autoclave, curing distortion is a common phenomenon in composite wall structures. This distortion not only affects the mechanical properties of the wall panel structure [4] but also hinders the assembly of components [1]. Hence, effectively mitigating the distortion of large-scale and complex cross-section composites has become a focal point and a challenging area of research [5].

Currently, the primary methods for mitigating distortion during the manufacturing of composites in an autoclave include the traditional trial-and-error method [6], the optimized design process parameters method [7,8,9,10,11,12], the mold profile compensation method [13,14,15,16,17], the hot sizing process method [18,19], and other control methods [20]. The traditional approach relies on experience and a trial-and-error process. It involves repeated adjustments and compensations of curing process specifications and mold profiles to control the degree of distortion. However, the use of traditional trial-and-error methods significantly increases costs for the production of composite wall panel structures. Despite the cost implications, this traditional method is still widely employed for large-scale composite components manufactured across various companies.

Thanks to the rapid advancements in finite element technology, we now have favorable conditions for delving deeply into solving the problem of distortion in composite structural parts, particularly in optimizing process parameters. Ren et al. [21] have studied the 3D woven composite process, developing process analysis agent models and optimizing process parameters. Their results demonstrate a reduction in residual strain and process cycle time with the use of optimized parameters. Manjusha et al. [22] have employed FBG sensors to monitor the curing process for changes in fiber volume within the resin matrix and composite material. Based on monitoring results, they have optimized the curing process parameters. Their findings reveal a reduction in residual stresses, process costs, and process cycle times within the interior of the composite component.

Nele et al. [23] conducted a study on the hot press molding process of thermoset resin matrix composites. They found that the two primary factors influencing laminate thickness, fiber percentage, and pore volume are external pressure and the timing of pressurization. Optimizing the timing of pressurization was effective in achieving the objective of reducing process costs and cycle times. However, when using the process parameters optimization method, a challenge arose in efficiently mitigating induced distortion for large-size and complex composite structures. In the literature [3,4,24,25,26,27], various approaches have been explored to predict and control distortion in composite components. Modifying the manufacturing mold profile is one method employed to manage component distortion. Despite extensive research, achieving perfect accuracy in predicting distortion, especially in large-scale and structurally complex cross-section composite components, remains challenging. Inaccurate distortion predictions can lead to time-consuming and costly redesign and rework of composite moldings [26]. Additionally, the maximum compensation amount significantly influences the accuracy of mold profile compensation, which, in turn, impacts the forming accuracy of the parts.

In the literature [18,19], the hot sizing process is described as a method in which a component is placed onto a mold that conforms to its shape under an external load, heated to a high temperature for a specific duration, and then unloaded to correct the profile of the composite material component. Liu et al. [24] investigated the fundamental principles of the hot sizing process for composite materials and conducted experimental studies on small composite structural components. The hot sizing process can partially modify the shape of composite components. However, it requires the design of specialized tooling, increases manufacturing costs, and has a detrimental effect on the strength and stiffness of the composite component.

In summary, the existing methods for mitigating curing distortion have limitations when applied to large-scale and complex cross-section composite structures. These limitations stem from the reliance on prediction accuracy, the associated high manufacturing costs, and the less-than-ideal effectiveness of distortion control. Therefore, we investigate the limitations of previously discussed control methods and present a refined approach to manufacturing composite parts.

This paper presents a global compensation method designed for large-scale and complex cross-section composite structures, addressing the challenge of distortion control in the autoclave process of composite wall panel structures. The approach involves establishing a coordinated model that links the refinement of design curing process parameters and the mold profile compensation method. A coordinated model simulation approach has been developed for large-scale, complex cross-section composite wall panel structures, predicting the final geometry of the wall panel structure. The study investigates the influence of heating, holding, and cooling times on distortion. To validate the method’s effectiveness, full-scale curing experiments were conducted using the autoclave process with a large-scale, complex cross-section composite wall structure measuring 1700 mm × 2000 mm. The refined manufacturing process achieves high-quality results for large-scale and complex cross-section main load-bearing composite structures, offering a theoretical foundation and practical value for the application of composites in the aerospace industry.

## 2. The Global Compensation Method and Coordinated Model

Due to irreversible curing distortion upon demolding, which fails to meet engineering’s practical requirements [28,29], existing methods struggle to effectively suppress distortion in thermoset composites processed in autoclave, especially for large-scale complex cross-section components. Achieving high-precision modeling with these methods remains challenging. Recognizing the limitations of current distortion control methods, this paper introduces a collaborative approach based on the global compensation of composite structural components. Central to this approach is the acquisition of the global compensation amount. The following section outlines the modeling process for the control method and the procedure for obtaining the global compensation quantity, known as the collaborative model.

**Figure 1:**
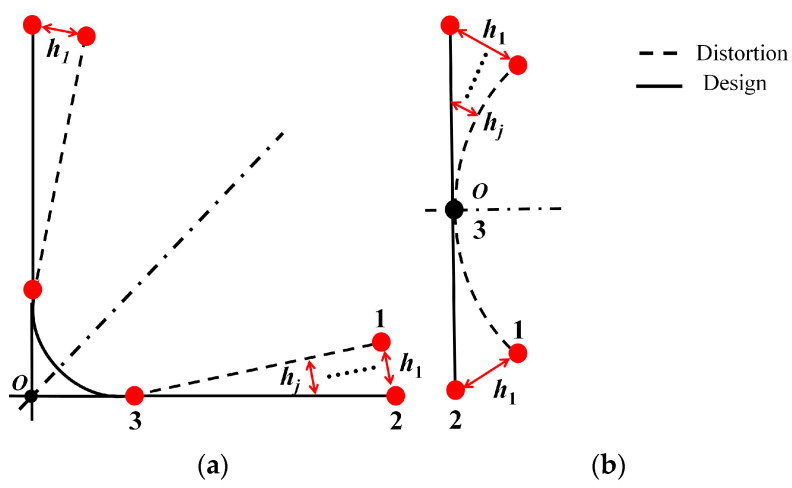
The theoretical analysis of the coordination model (**a**) curvature sample; (**b**) plat sample.

First, we design the optimal curing process parameters to mitigate the curing distortion of the composite component while adhering to cost constraints. Next, we establish coordinated models, as illustrated in Figure 1a,b. These models consist of the theoretical design model’s inner surface and the outer profile of the component after curing and distortion. On the theoretical profile, we select ‘n’ nodes, denoted as *n_i_* (*i* = 1~*n*). Since the grid comprises four-node elements, each element’s area is represented by the element weight ratio *S_i_*. For every four-node tetrahedral mesh element (or hexahedral mesh element), we set a projection distance along the normal direction, denoted as *h_j_* (*j* = 1~*n*), from point 1 to point 2.

We assume that there are ‘*m*’ grids on the surface of the simulation component model. The model for calculating the weighted average of cured distortion in the simulated composite component model is represented by the following:

(1) $$D_{av}=\frac{1}{m}\sum_{i=1}^{m}S_{i}\times \frac{1}{n}\sum_{j=1}^{n}h_{j}$$

where *S_i_* stands for the area of the *i*-th grid on the surface of the simulation component model, and *h_j_* represents the distance from the *j*-th node on the grid of the simulation component model to the manufacturing profile of the theoretical mold model along the normal direction.

The model for calculating the root mean square distortion value in the simulated composite component model is as follows:

(2) $$D_{msr}=\sqrt{\sum_{j=1}^{n}h_{j}^{2}}$$

where *h_j_* denotes the distance from the *j*-th node on the grid of the simulation component model to the manufacturing profile of the theoretical mold model along the normal direction.

We obtain the mean value of weighted distortion, the root mean square distortion, the minimum distortion value, and the maximum distortion for group *k*. To select the smaller value for global compensation, we follow this method: first, we arrange the weighted distortion averages into *k* groups, from smallest to largest. Then, we select the first *k*/2 + 1 groups of weighted distortion averages and find their corresponding root mean square distortion values. Next, we arrange the *k*/2 + 1 sets of root mean square distortion values obtained in the first step from smallest to largest and choose the front *k*/4 + 1 groups, finding their corresponding minimum distortion values. We then organize the *k*/4 + 1 groups of minimum distortion values obtained in the second step from smallest to largest, select the front *k*/8 + 1 groups, and find the corresponding maximum distortion values. Finally, we arrange the *k*/8 + 1 groups of maximum distortion values from largest to smallest and select the very smallest of the distortion maxima as the smaller value for global compensation.

**Figure 2:**
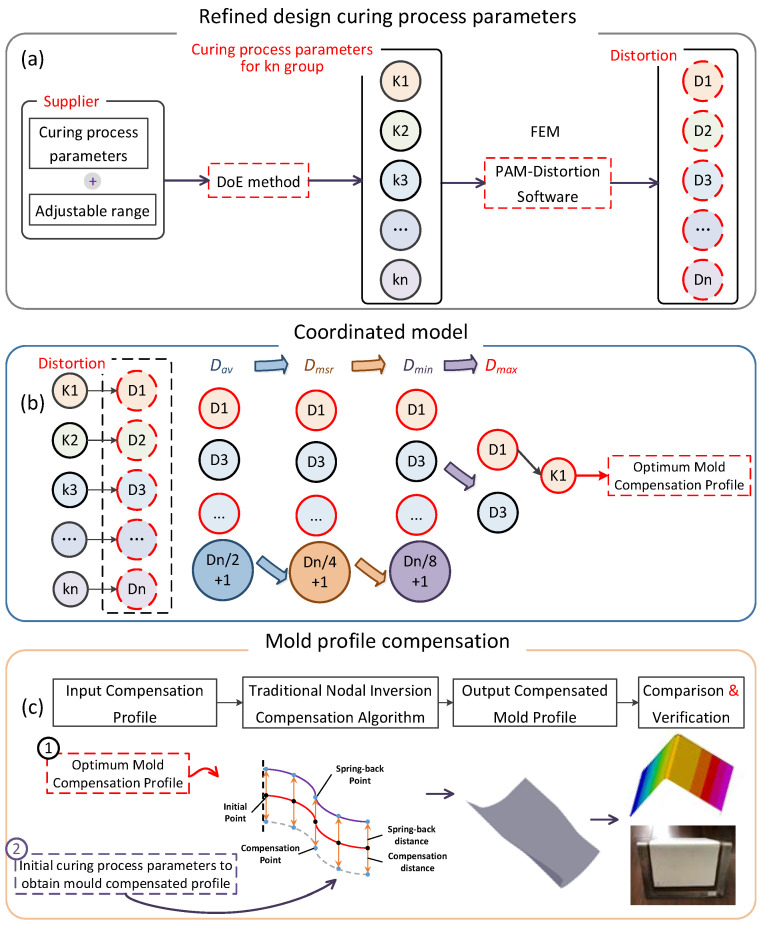
The optimal compensation profile obtainment and mold compensation process. (**a**) Refined design curing process parameters; (**b**) coordinated model; (**c**) mold profile compensation.

The process of obtaining the optimal compensation profile and mold compensation is depicted in Figure 2. Firstly, we conduct a refined design of the curing process parameters, as shown in Figure 2a. Based on the curing process parameters and their adjustable ranges provided by the supplier, multiple combinations of process parameters (k1, k2, k3, …, kn) were designed using the Design of Experiments (DoE) method. Utilizing PAM-Distortion (2014) software, simulation analyses were conducted to obtain the curing deformation values (D1, D2, D3, …, Dn) corresponding to different combinations of process parameters. In the simulation analysis, the curing deformation values (D1, D2, D3, …, Dn) were obtained for the respective combinations of process parameters (k1, k2, k3, …, kn). Subsequently, *D_av_* and *D_msr_* were calculated using Formulas (1) and (2). A four step process of ranking and selection was performed to ultimately obtain a set of process parameters and their corresponding cured component distortion results, as shown in Figure 2b. The obtained cured component deformation results serve as the optimal mold compensation profile. Finally, the optimal compensation profile and the mold surface to be compensated for are imported into the software, and the mold surface is compensated for based on the traditional nodal reverse compensation algorithm. The compensated mold surface is then outputted, and the compensation effect is compared and analyzed using both finite element methods and curing experiments for verification, as shown in Figure 2c.

This marks the completion of the coordinated model. Finally, the smaller value for the global compensation of the mesh surface of the simulated component model corresponds to the manufacturing mold profile obtained using compensated finite element (2014) software.

## 3. Materials, Systems, and Properties

The prepreg material system for braiding consists of T800 carbon fiber and 603A epoxy resin [30]. Each single layer of prepreg has a thickness of 0.2 mm, and it achieves a fiber volume rate of 56%. For filling materials, we use Polymethacrylimide (PMI) foam with a density of 110 kg/m^3^ and an elastic modulus and strength of 135 MPa and 2.2 MPa, respectively. Additionally, we employ ortho-hexagonal Nomex paper honeycomb structures with a density of 32 kg/m^3^ and equivalent elastic modulus and strength values of 623 MPa and 60 MPa, respectively. The composites are cured at 165 °C, with a heating time of 5.6 h, a holding time of 4 h, and a cooling time of 6.25 h, resulting in an initial total curing time of 15.85 h. The curing process is carried out under a pressure of 0.3 MPa. For thermo-chemical parameters and fundamental mechanical properties of the T800 carbon fiber/epoxy composite, please refer to Table 1 and Table 2, respectively.

*ρ_c_* represents the composite material density, which can be calculated as a weighted average of volumetric content from the density of resin and fibers, indicated with the symbols *ρ_m_* and *ρ_f_*, respectively; *C_p,c_*. denotes the composite-material-specific heat, which can be determined as a weighted average of weight content from the specific heat of fibers and resin. The thermal conductivity of a single unidirectional ply can be defined by two terms: the conductivity along fibers, K*_l_*, and the perpendicular conductivity, *K_t_*. The resin total reaction heat is *H_r_*, *E* represents the activation energy, *A* the frequency factor, and *m* and *n* the reaction orders. *λ* is a parameter characteristic, T_g∞_ is the maximum glass transition temperature, and T_g0_ the minimum temperature.

### 3.1. Curing Kinetics Model

**Figure 3:**
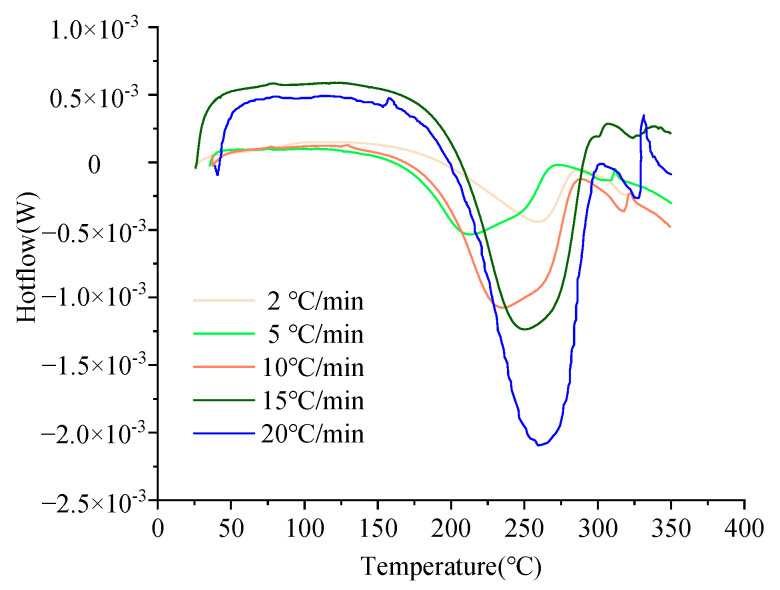
DSC curves of 603A resin at different heating rates.

The curing kinetics model illustrates the quantitative relationship between the resin’s cure rate, the degree of cure, and the temperature. We conducted five sets of DSC experiments with varying rates of warming. These experiments used heating rates of 2, 5, 10, 15, and 20 °C/min, with each group repeated once. Figure 3 presents the results of the dynamic DSC scan tests at different ramp rates. As the temperature increase rate escalates, the curing reaction rate of the 603A resin decreases, and the time required to reach peak heat flow increases.

The starting temperature (*T*_i_), peak temperature (*T_p_*), termination temperature (*T_f_*), and the total amount of heat released after the complete reaction (*H*_u_) for the curing reaction of 603A resin at different temperature rise rates (*β*) are presented in Table 3. The average total heat release after the complete curing reaction of 603A resin is 403.5 kJ/kg.

**Figure 4:**
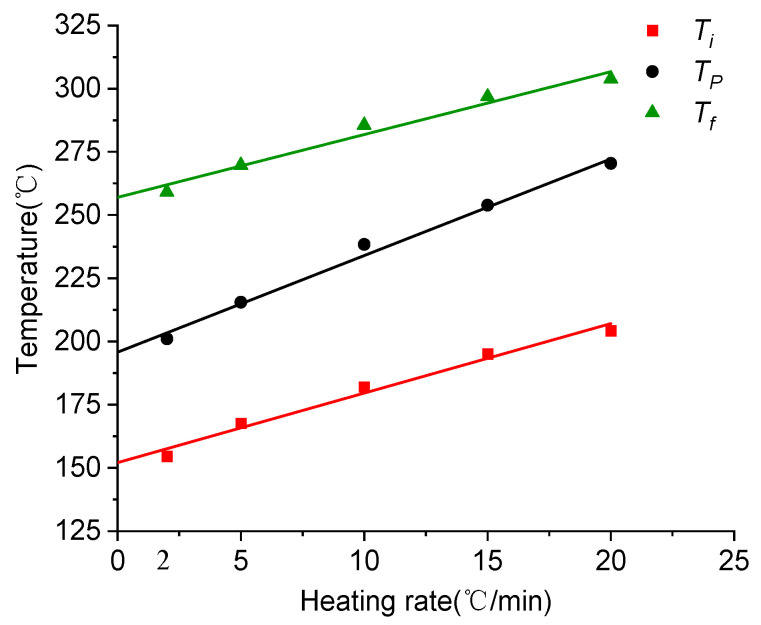
*T*-*β* extrapolation diagrams for 603A resin.

Based on the characteristic temperature of the exothermic peak of the DSC curve at different ramp rates, the *T*-*β* extrapolation method was used to determine the curing process temperature of 603A resin, as shown in Figure 4. The curing process temperatures for 603A resin include 151.3 °C for gelation, 193.9 °C for curing, and 253.6 °C for post-treatment. In the actual curing and molding process, other factors must be taken into consideration when determining the curing temperature for the component. For instance, when the skin is co-cured with the frame beam as a whole, including a sandwich layer, this can reduce the required curing temperature and extend the holding time.

In summary, the kinetics of 603A epoxy resin curing are modeled by the following equation:

(3) $$\frac{d\alpha }{dt}=9930.76\exp\left(\frac{-7220.57}{T}\right)\alpha^{0.36}{\left(1-\alpha \right)}^{1.25}$$

where *R* represents the universal gas constant, *α* the cure degree, and *T* the temperature.

### 3.2. Heat Transfer Model

In an autoclave, air is heated to control the temperature of the mold and the component during the curing process through solid heat transfer. The temperature transfer and distribution within composite components are regarded as a nonlinear endothermic problem. This is based on Fourier’s law of heat transfer and the law of conservation of energy, leading to the establishment of the following equation as the governing equation for heat transfer and distribution in an autoclave [31,32,33]:

(4) $$\frac{\partial }{\partial x}\left[k_{x}\frac{\partial T}{\partial x}\right]+\frac{\partial }{\partial y}\left[k_{y}\frac{\partial T}{\partial y}\right]+\frac{\partial }{\partial z}\left[k_{z}\frac{\partial T}{\partial z}\right]+\overset{\cdot }{Q}=C_{T}\frac{\partial T}{\partial t}$$

where *T* stands for temperature, *C_T_* represents the specific heat of the material, and *k_x_*, *k_y_*, and *k_z_* are the coefficients of heat transfer along the *x*, *y*, and *z* directions.

$\overset{\cdot }{Q}$ is the rate of heat generation related to the exothermic nature of the curing reaction and is expressed as the following equation [34]:

(5) $$\overset{\cdot }{Q}=\left(1-V_{f}\right)H_{r}\frac{d\alpha }{dt}$$

where *t* is time, *V_f_* is the fiber volume content, *H_r_* is the total exothermic reaction heat per unit mass of resin cured, and *α* is the degree of cure.

### 3.3. Stress–Strain Constitutive Models

**Figure 5:**
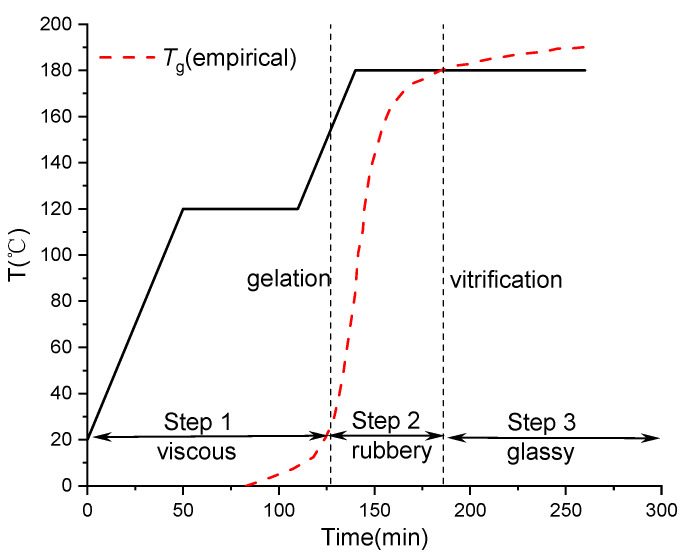
Typical three stages of the curing process of composites.

Thermoset composites undergo curing reactions during the molding process, with the degree of cure of the resin increasing with temperature and time. The phase state transitions through three stages: viscous flow state, rubbery state, and glassy state [35,36], leading to significant changes in mechanical properties, as depicted in Figure 5.

## 4. L-Shaped Composite Laminates

### 4.1. Numerical Simulation Analysis

Numerical simulations were conducted to predict the curing distortion of the composite wall panel structure using PAM-COMPOSITES finite element analysis (2014) software [29,37]. This software offers numerical simulations of temperature and curing degree fields, as well as residual stresses and induced distortions in composite components through the use of a thermo-chemical module and a thermo-mechanical module, respectively. The numerical simulation type definitions are configured for curing, during curing, and after curing, in that order.

**Figure 6:**
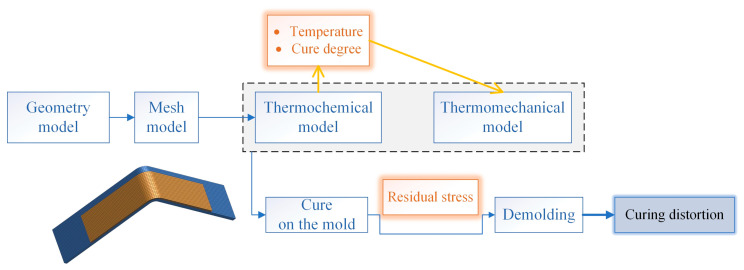
Schematization of the simulation steps.

The simulation was formed by three steps, as reported in Figure 6: first of all, both the degree of cure and the temperature trends were determined for each node of the calculation mesh by means of the thermo-chemical module; then, the development of the residual stresses in the laminate still blocked on the mold was calculated by the thermo-mechanical model, starting from the results obtained in the previous simulation step; finally, the distortions and the spring-in were determined through the thermo-mechanical module, in which the mold removal and the consequent stress relaxation were simulated. The material properties required for the calculation of the thermo-chemical step are reported in Table 1. The material properties required for the calculation of the thermo-mechanical step are reported in Table 2.

**Figure 7:**
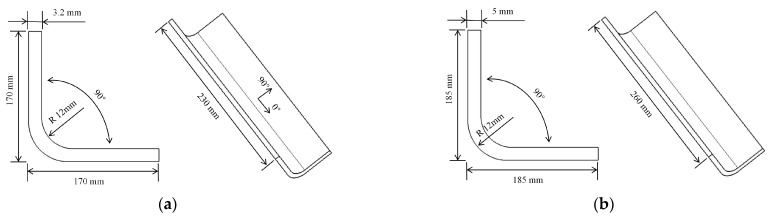
Skeleton of (**a**) part and (**b**) mold.

L-shaped components and mold models for manufacturing are depicted in Figure 7a and Figure 7b, respectively. The manufacturing process involves the use of a male mold form for creating composite components. The mold material used for manufacturing is *Q*235. The dimensions are as follows: the width (*t*) is 60 mm, the thickness (*H*) is 10 mm, and the corner radius (*R*) is 12 mm. The L-shaped component has a width (*t*_1_) of 170 mm, a thickness (*H*) of 3.2 mm, and a corner radius (*R*_1_) of 12 mm. The composite L-shaped component consists of 16 layers, with each prepreg having a thickness of 0.2 mm, resulting in a total thickness of 3.2 mm. The lay-up design is [0/45/0/45/45/45/0/45/0]_s_ for the structural element.

The temperature distribution within the composite part plays a crucial role in determining the degree of curing inside the component. It is essential to take into account the heat released during the curing reaction of the resin matrix in the heat conduction process of the composite curing procedure.

### 4.2. Grid Independence Verification

**Figure 8:**
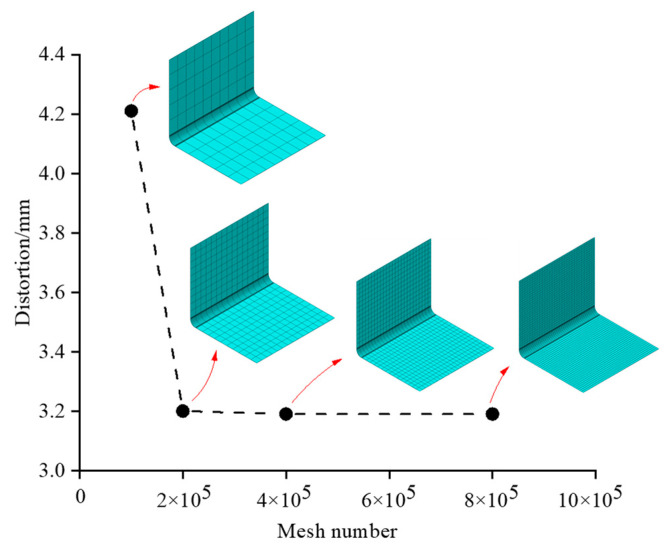
Maximum induced distortion of composite.

To ensure that the number of meshes is independent of the numerical simulation results, the relationship between the maximum curing distortion and the number of meshes of the composite L-shaped structural member is calculated and summarized, as shown in Figure 8. The results of the models in different mesh numbers, from 100,000 to 200,000, with a decrease of 1.11 mm, are compared. The results of the models in different mesh numbers, from 200,000 to 800,000 with a decrease of 0.01 mm, are compared [38]. The models with mesh numbers from 200,000 to 800,000 all have consistent results. In conclusion, to ensure the accuracy of numerical simulation results while saving computational resources and improving computing efficiency, it was determined that the number of mesh elements for the composite L-shaped structural elements should be approximately 200,000.

### 4.3. Refined Design of the Curing Process Parameters

Based on the initial curing process curve provided by a unit, the parameters for the preferred combination were selected as heating time (*T*_h_), holding time (*T*_k_), and cooling time (*T*_c_). These parameters were used to establish better levels of parameter intervals, as indicated in Table 4. Using the design methodology of the DoE experiment, multiple sets of coupled process parameters were obtained, as shown in Table 5.

The calculation of the weighted average and root mean square values of distortion is essential in the overall concept of the coordinated manufacturing process scheme. The weighted mean, root mean square, maximum, and minimum values of distortion for each set of curing process parameter combinations, as proposed in this paper, are presented in Table 6.

Following the coordinated model, a set of coupled parameters for the curing process corresponding to the compensation profile of the manufacturing mold with the highest global compensation value is obtained, as shown in Table 7. The final manufacturing mold profile is then determined by utilizing the mold profile associated with the lowest global compensation value, and this mold profile is further adjusted using DynaForm’s SCP (5.9.4) software module.

### 4.4. Experimental Analysis Model

The detailed parameters of the autoclave used for this curing experiment are provided in Table 8. The composite material used is T800 carbon fiber/603A epoxy resin (Nanjing Julong Composite Technology Co., Ltd., Nanjing, China), and the lay-up is consistent with that in the numerical simulation analysis model. The specimen should be manually laid out and then cured using an autoclave [37,38]. Experimental aids such as RP3 release cloth, breathable felt, polyimide barrier film, L500Y vacuum bags, and K-type thermocouples were utilized. The K-type thermocouples were strategically positioned in both the uppermost and middle layers of the mold surface and the composite component. This placement allowed for the continuous monitoring of temperatures in order to prevent temperature disparities. The temperature trends recorded by all thermocouples were found to be in alignment with the thermal cycles outlined in the prepreg data sheet. Temperature control was primarily based on the mold’s temperature. Following the test’s cold pressing phase, the temperature was gradually increased and halted when it reached 60 °C. As the process advanced, the temperature decreased in sync with the furnace, and the mold’s temperature was maintained at or below 40 °C when the autoclave was discharged. It is important to note that during the curing experiment, the air temperature within the autoclave must not exceed 200 °C while raising the temperature.

**Figure 9:**
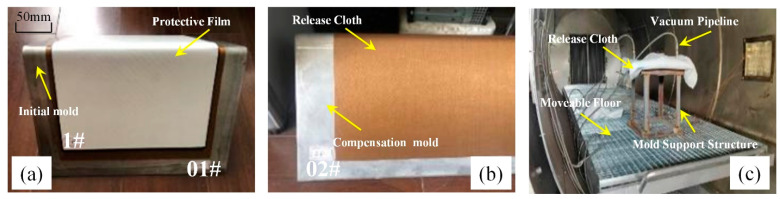
Autoclave process of curing experiment: (**a**) initial mold and uncured composite components; (**b**) compensated manufacturing die obtained in this paper; (**c**) field diagram of curing test.

As shown in Figure 9a, it is the initial mold (01#). As depicted in Figure 9b, it is the compensated mold (02#). First, a release cloth is applied to the mold surface. Then, the pre-cut prepregs are layered in the desired stacking sequences. After the prepregs are laid, a layer of release cloth, a separator film, and a breathable felt are sequentially applied. Next, the vacuum pipeline is connected to pressurize the composite component for curing, as shown in Figure 9c. Figure 9c includes the vacuum pipeline used to provide pressure, the support plate structure for supporting the mold, and the release cloth for easy mold release. Once curing is complete, the composite component is separated from the mold to obtain the cured sample.

**Figure 10:**
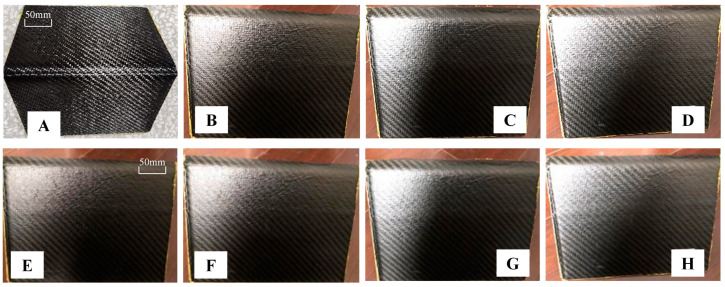
Mold curing samples: (**A**–**D**) 01# mold curing sample; (**E**–**H**) 02# mold curing sample.

In this article, the sizes of the sample and mold are consistent with those shown in Figure 7. As shown in Figure 10A–D, four samples (1#, 2#, 3#, 4#) were produced using the original mold (01#). As depicted in Figure 10E–H, four samples (1#, 2#, 3#, 4#) were produced using the compensated mold (02#).

## 5. Composite Wall Panel Construction

**Figure 11:**
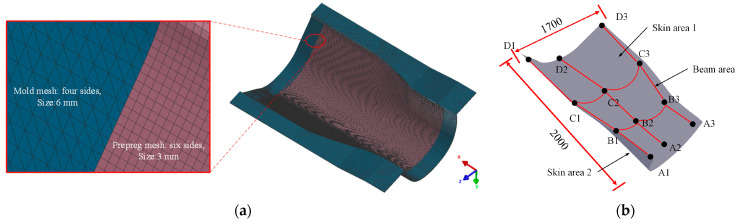
Skeleton of wall panel: (**a**) mesh of wall panel; (**b**) T800 carbon fiber/603A epoxy composite.

The T800 carbon fiber/epoxy composite wall panel structure possesses specific dimensions and a complex curved surface with varying curvature, as depicted in Figure 11a. Along the length of the wall panel structure, several composite bars and additional auxiliary components are distributed. The space between the upper (Skin area 1) and lower (Skin area 2) skins is filled with PMI foam and paper honeycomb. The wall panel structure is divided into three sections: the front, mid-transition, and back-end areas, each with its unique lay-up configuration as outlined in Table 9. A mesh model of the composite wall panel structure is illustrated in Figure 11b. The curing process curve entails heating, maintaining, and cooling for 5 h, 3 h, and 4 h, respectively, totaling 12 h (originally, the total curing time was 16.8 h).

## 6. Results and Discussion

### 6.1. Simulation and Experimentation of L-Shaped Composite Laminates

#### 6.1.1. Analysis of Temperature Field Mechanisms

**Figure 12:**
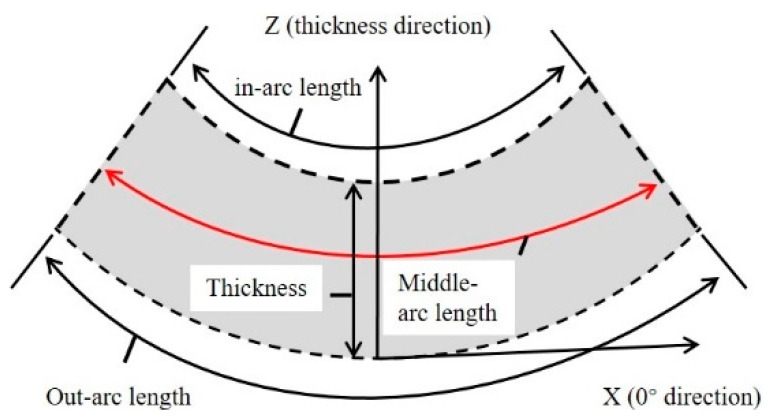
In–middle–out arc length degree of cure.

**Figure 13:**
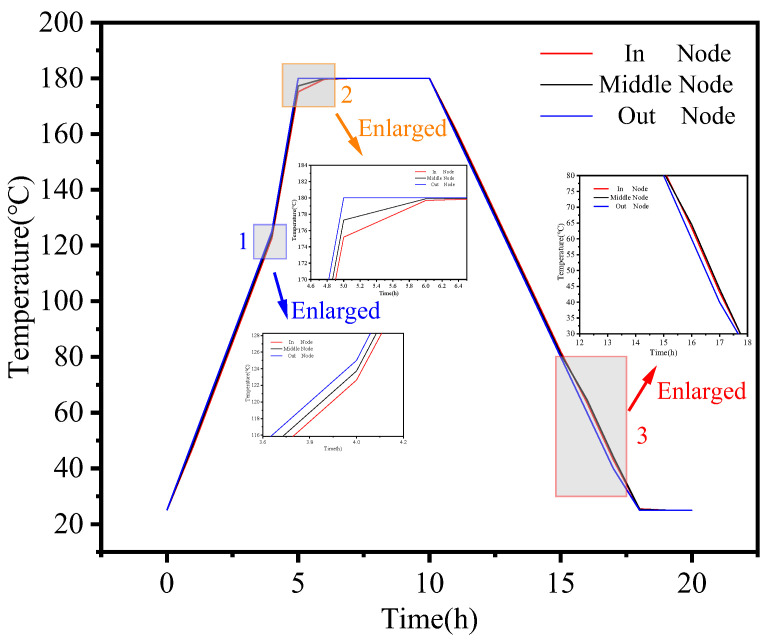
In–middle–out arc length temperature and initial temperature.

To provide a more efficient and clear explanation of the numerical simulations and experimental results, we have established a schematic diagram illustrating the in–middle–out plane distribution of the composite member, as displayed in Figure 12. It is worth noting that there exists a noticeable temperature gradient in the temperature values between the outermost, middle, and innermost layers of the composite component during heating phase interval I, as depicted in Figure 12 and Figure 13. As part of the manufacturing process, we have extracted local zooms to reveal the temperature trends among different layers during interval II of the heating phase. In the enlarged diagram, it becomes evident that there is a temperature inflection point at point 1, resulting in a sharp increase in temperature along the in–out plane. This occurrence can be attributed to the exothermic phenomenon that takes place when the resin matrix reaches a specific temperature.

In stage IV, as the manufacturing process advanced, it became evident that the nodal temperature of the outer surface was notably lower than the temperature values of the inner and intermediate layers for the same curing time, as depicted in Wireframe 3. The intermediate layer’s temperature was relatively high during this stage. This temperature distribution arises from the fact that the internal temperature of the composite member cannot cool down uniformly during the cooling process [38]. In summary, the numerical simulation results presented in this paper align with the results obtained from the curing experiments, demonstrating the accuracy of the numerical simulation model.

As shown in Figure 9 and Figure 10, and Table 10, a comparison between the numerical simulation results and the curing experimental results reveals a difference of 3.2 mm and 3.181 mm, respectively, with an error value of 0.59% when using 01# (the initial design mold surface) for curing. This underscores the reliability of the numerical simulation results presented in this paper. When 02# (the mold surface obtained by the method in this paper) was used for curing the mold, a comparison between the numerical simulation results and the curing experimental results (as shown in Table 10) indicated an error of only 5.1%. This demonstrates the high level of accuracy achieved through the numerical simulation. Furthermore, by comparing the results of curing with molds of different sizes for smaller L-shaped composite laminates using 01# and 02#, this method not only ensures the accuracy of composite component manufacturing but also reduces the autoclave process curing time by 4.85 h, resulting in significant cost savings in the manufacturing process.

#### 6.1.2. Effect of Temperature Heating Time on Residual Stress and Induced Distortion

**Figure 14:**
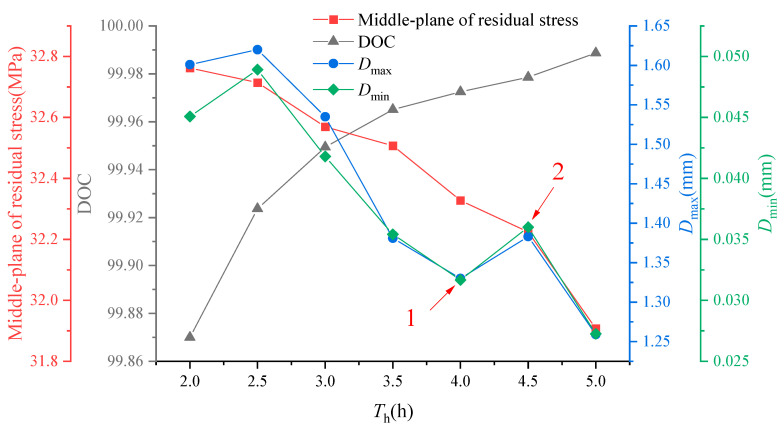
The influence of heating times on residual stress and curing distortion.

With an increase in heating time, the overall resin cure of the composite component gradually approaches 100% upon the completion of the manufacturing process. The residual stress values in the intermediate layers of the composite components exhibit a gradual reduction as curing time increases, as depicted in Figure 14. This reduction further suggests that longer heating times lead to lower residual stresses within the component. These findings align with the conclusions drawn in the previous section. Moreover, the maximum and minimum values of induced distortion follow a similar pattern of increase then decrease, followed by an increase and ultimately decrease as heating time increases [39,40]. By comparing points 1 and 2 in the figure, it becomes evident that locally optimal solutions exist for the combination of manufacturing process parameters for composite components of varying structural dimensions.

#### 6.1.3. Effect of Temperature Keeping Time on Residual Stress and Induced Distortion

**Figure 15:**
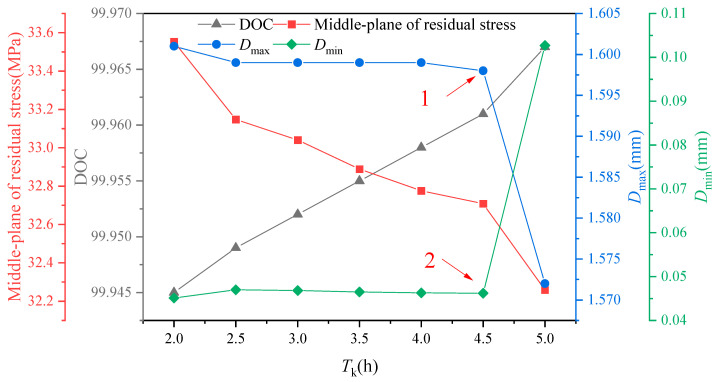
The influence of keeping times on residual stress and curing distortion.

According to Figure 15, the residual stresses in the middle layer of the component exhibit a gradual decrease. It is evident from the variations in residual stresses that longer holding times have a favorable impact on the curing quality of the composite component. Furthermore, the maximum value of induced distortion follows a gradual decrease, with a sharp decline occurring at point 1 (at 4.5 h), while the minimum value of induced distortion also experiences a sharp increase at point 2. The abrupt changes at points 1 and 2 collectively suggest that there exists an optimal value for the effect of holding time on induced distortion within a localized interval.

#### 6.1.4. Effect of Temperature Cooling Time on Residual Stress and Induced Distortion

**Figure 16:**
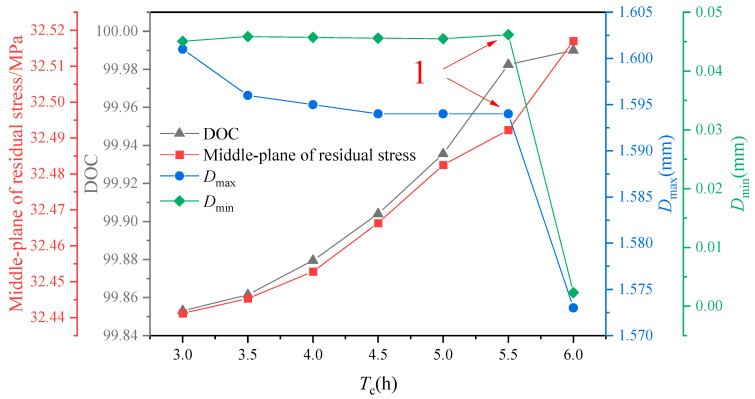
The influence of cooling times on residual stress and curing distortion.

As shown in Figure 16, there is an increasing trend in the residual stress in the middle layer of the component as the cooling time increases. This trend suggests that a longer cooling time negatively impacts the molding process of the composite component. Additionally, both the maximum and minimum values of induced distortion exhibit a gradual decrease, with a sharp decline in the maximum value of induced distortion at point 1 in the illustration. An analysis of the combined effect of cooling time on the curing process of the components reveals opposite patterns of change in the optimum values of residual stress and induced distortion. This indicates that it is not possible to consistently improve manufacturing accuracy by increasing the cooling time during the manufacturing process, as it can have varying effects on different aspects of the composite component. The predicted stress level is consistent with that in [41].

### 6.2. Simulation of and Experimentation on Composite Wall Panel Construction

**Figure 17:**
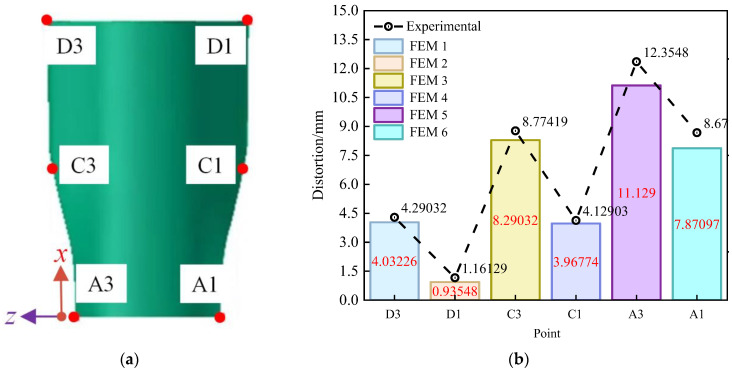
Distribution of measurement points and comparison of simulation and experimental results (initial): (**a**) distribution of measurement points; (**b**) comparison of simulation results and test results.

Following the cooling and demolding process, the center of the composite wall panel structure is pressed against the tooling with a pressure bag. The distance between the wall panel elements and the manufacturing mold is measured at positions A1, A3, C1, C3, D1, and D3, as shown in Figure 17a. The numerical analysis method demonstrates good agreement with the test results, with a maximum error of 17.4% and an average error of 8.6%, as depicted in Figure 17b for the specific location comparison. Notably, there are significant prediction errors at positions C1 and C3. This can be attributed, in part, to the additional stress field between the prepreg layers resulting from forced pre-laying during the test. This non-uniform stress field within the part during the forming process leads to lateral distortion. Furthermore, the presence of local eddy currents resulting from convective heat transfer in the autoclave can, in practice, create disparities between the actual temperature field and the predetermined temperature field. This, in turn, has a cascading effect on the thermo-mechanical properties of the prepreg, potentially leading to excessive distortion. Moreover, the equations intentionally used in this paper do not incorporate the impact of temperature on the viscoelastic properties of the material, which can lead to deviations at other points in the analysis.

**Figure 18:**
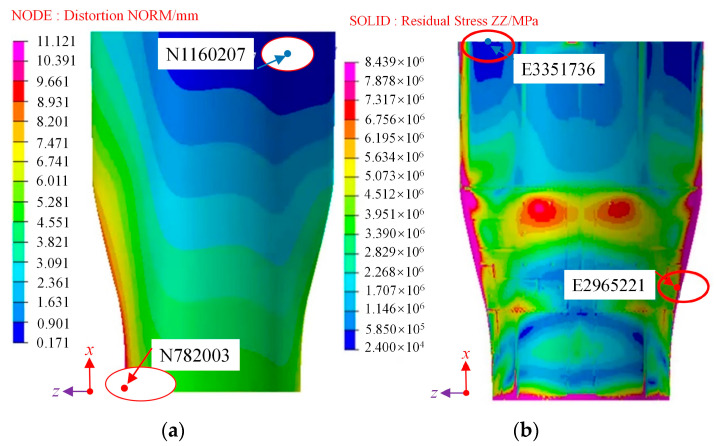
The clouds following demolding: (**a**) distortion; (**b**) residual stress.

The clouds following demolding, as obtained through the numerical analysis method, are presented in Figure 18. The induced distortion of the wall panel structure is primarily characterized by a rebound effect, with a tendency to shrink inwards. The curing distortion demonstrates a gradual decrease from the two endpoints toward the centrosymmetric position, with the smallest induced distortion occurring at the centrosymmetric point of the wall panel structure. The maximum distortion observed in the wall panel structure measures 11.121 mm and is situated at the corner point of the short side in the direction of the axis of symmetry (N782003), as shown in Figure 18a. Conversely, the minimum distortion is 0.171 mm, located near the corner point of the short side in the direction of the axis of symmetry (N1160207). Along the axis of symmetry, the spring-back increases progressively from the long side to the short side.

Residual stresses are a primary factor contributing to the spring-back of composite components, and a residual stress distribution for the wall panel structure is presented in Figure 18b. Residual stresses are notably higher at the more distorted sides and shorter edges, with a difference of approximately 7 MPa when compared to the less distorted longer edges. This difference is attributed to the thermal distortion, which induces chain breaking and rearrangement of the molecular chains within the epoxy resin. Increased distortions lead to more significant changes in the molecular chains, further enhancing the physical cross-linking between them. These changes and the cross-linking processes are responsible for the buildup of residual stresses in the prepreg during the manufacturing process.

Furthermore, increased distortion decreases the contact area between the epoxy resin and the carbon fibers, resulting in reduced local wettability of the prepreg. This, in turn, leads to non-uniform macroscopic wettability of the prepreg and ultimately results in the concentration of residual stresses. These residual stresses directly impact the spring-back of the deformed component after manufacturing. The spring-back of the component is influenced by a combination of external force fields, such as demolding forces, and internal force fields, like residual stresses. Once again, this paper’s numerical simulation method has been validated for its accuracy.

**Figure 19:**
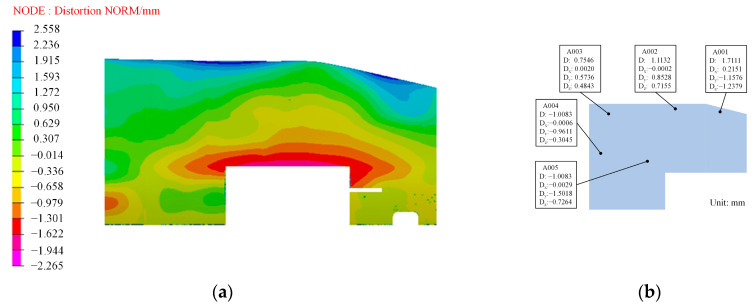
The resulting cloud of deviations: (**a**) the difference between the actual cured finite wall panel structures and the theoretical distortion; (**b**) experimental results.

First, the optimal compensation surface was obtained based on the method proposed in this paper. Next, the initial mold surface was compensated for to obtain the compensated mold, using the same compensation method as previously described. Subsequently, the curing simulation of the wall panel structure was performed once more using the surfaces molded according to the method outlined in this paper. The resulting cloud of deviations, representing the difference between the actual cured finite wall panel structures and the theoretical distortion, is presented in Figure 19a. It is important to note that, during the comparison, we only extract the lower surface of the cured component’s deformation result, specifically, the surface where the film is applied.

Based on the observations in Figure 17, the induced distortion of the wall panel structure produced through this method exhibits a high degree of uniformity. However, it is important to note that the lower surface of the distorted composite wall panel structure is offset from the upper surface of the originally molded panel, and the actual measurements of the lower surface are displayed in Figure 19b. The experimental results confirm that the coordinated manufacturing process facilitates the cost-effective and highly accurate production of large and complex wall plate structures.

The maximum relative error (|simulated value − experimental value|/|experimental value|) is 19.17% when comparing the measured values of the test deviations at the different selected points with the numerically simulated values. It is worth noting that engineering acceptance requirements typically do not exceed a 20% deviation from the actual measured and simulated analysis results. These outcomes are detailed in Table 11. Furthermore, this paper validates the accuracy and efficiency of the two-level coordinated manufacturing process.

The primary sources of error in the analysis are as follows:

(1) Composite components are significantly influenced by the preparation of prepreg. In cases wherein composite components are thin, human factors become non-negligible when manually laying them.
(2) Errors may be introduced during the scanning process and data processing when measuring wall panel structures.
(3) Numerical simulation analysis can only approximate various settings to the actual conditions but may not precisely replicate them, leading to discrepancies and errors.

## 7. Conclusions

The purpose of the present work is to reduce the curing distortion of large-scale and complex cross-section composite structures. The paper is divided into three parts: in the first one, a coordinated model for the refined design of manufacturing process parameters and mold profile compensation is developed. A global compensation method is proposed by introducing an optimal profile; in the second part, the method is used for investigating L-shaped composite structures and composite wall panel construction with large-scale and complex cross-sections, employing both numerical simulation analysis and experimental analysis. The quantitative discussions about the influence mechanisms of different process parameters, including heating, holding, and cooling times, are accomplished. Some conclusions can be extracted from the results and discussions:

(1) The control of the curing distortion of composite with large-scale and complex cross-sections using the proposed method, by introducing an optimal profile, greatly improves the forming accuracy. The proposed model balances the forming time and accuracy, which is urgently needed in practice.
(2) The maximum curing distortion of the structure was restrained from 11.121 mm to 1.711 mm after manufacturing a large complex composite wall panel structure using a global compensation method. The total manufacturing time was reduced from 15.85 h to 11 h by fine-tuning the design of the curing process parameters. This refinement also led to an improvement in the overall uniformity of the distribution of curing distortions in this structure, decreasing it from 10.95 mm to 1.122 mm.
(3) The residual stresses in the middle layer of the fabricated parts exhibited a decreasing trend with increasing heating time and holding time. Conversely, an opposite trend was observed with an increase in cooling time.
(4) The global compensation method is suitable for the high-precision manufacturing of composite structural components in different mold profiles, structures, stacking sequences, and scales.

## Figures and Tables

**Table 1:** Thermo-chemical parameters of T800 carbon fiber/603A epoxy composite.

Thermal Parameter	Value	Curing Kinetic Parameter	Value
*ρ_c_*/(kg·m^−3^)	1580	*A* (s^−1^)	9930.76
*ρ_m_*/(kg·m^−3^)	1300	*E* (J·mol^−1^)	60,030
*Cp,c*_._/(J·kg^−1^·K^−1^)	946.36	*m*	0.36
*K_l_*/(W·m^−1^·K^−1^)	4.65	*n*	1.25
*K_t_*/(W·m^−1^·K^−1^)	0.45	*H*_r_ (J·kg^−1^)	483,000

**Table 2:** Mechanical parameters of T800 carbon fiber/603A epoxy composite.

Parameters	Rubber Value	Glass Value
*E_11_*/MPa	132,000	135,000
*E_22_* = *E_33_*/MPa	165	6500
*G_12_* = *G_13_*/MPa	44.3	4900.0
*G_23_*/MPa	41.6	3270.0
*μ*_12_ = *μ*_13_	0.346	0.300
*μ*_23_	0.982	0.450
*ε11th*/με/°C	0	0
*ε22th* = *ε33th*/με/°C	0	32.60
*ε* *_11_* *^cu^*	0	0
*ε_22_^cu^* = *ε* *_33_* *^cu^*	−0.048	0
DiBenedetto’s equation coeff.	*Value*	-
T_g0_/°C	7.1	-
T_g∞_/°C	250.0	-
*λ*	0.78	-

**Table 3:** Characteristic parameters of 603A resin DSC curves at different ramp rates.

No.	*β* (°C/min)	*T_i_* (°C)	*T_p_* (°C)	*T_f_* (°C)	*H_u_* (kJ/kg)
1	2	153.4	198.8	255.8	395.6
2	5	166.3	212.9	265.9	414.5
3	10	180.6	235.1	281.4	403.8
4	15	193.3	250.2	292.6	396.7
5	20	202.2	266.6	299.0	406.7

**Table 4:** Parameter adjustment range.

No.	Parameter	Low Level	High Level
1	*T*_h_	3	6
2	*T*_k_	2	5
3	*T*_c_	3	6

**Table 5:** Process parameter combination.

No.	*T*_h_/h	*T*_k_/h	*T*_c_/h	Total Time/h
1	6.0	2	6.0	14.0
2	3.0	5	3.0	11.0
3	3.0	2	6.0	11.0
4	3.0	2	3.0	8.0
5	6.0	5	6.0	17.0
6	6.0	5	3.0	14.0
7	6.0	2	3.0	11.0
8	3.0	5	6.0	14.0
9	2.5	3	3.0	8.5
10	3.0	3	3.0	9.0
11	3.5	3	3.0	9.5
12	4.0	3	3.0	10.0
13	4.5	3	3.0	10.5
14	5.0	3	3.0	11.0
15	3.0	2	3.0	8.0
16	3.0	2	3.5	8.5
17	3.0	2	4.0	9.0
18	3.0	2	4.5	9.5
19	3.0	2	5.0	10.0
20	3.0	2	5.5	10.5
21	3.0	2	6.0	11.0

**Table 6:** Main parameter values in the coordinated model.

Number	*D*_av_	*D*_msr_	*D*_max_	*D*_min_
1	19.189203	3.691516	1.601	−0.0661
2	19.641000	3.753058	1.620	−0.0489
3	18.375700	3.537211	1.535	−0.0648
4	16.512359	3.181012	1.381	−0.0607
5	15.863734	3.060563	1.330	−0.0611
6	16.538390	3.186050	1.383	−0.0602
7	14.975375	2.893519	1.259	−0.0607
8	19.189203	3.691516	1.601	−0.0661
9	19.183516	3.687550	1.599	−0.0639
10	19.188516	3.688653	1.599	−0.0641
11	19.178953	3.687030	1.599	−0.0644
12	19.176375	3.687156	1.599	−0.0646
13	19.167922	3.685578	1.598	−0.0647
14	19.588844	3.687904	1.572	−0.0230
15	19.189203	3.691516	1.601	−0.0661
16	19.134140	3.679510	1.596	−0.0648
17	19.125281	3.677971	1.595	−0.0650
18	19.116922	3.676536	1.594	−0.0651
19	19.114281	3.676124	1.594	−0.0652
20	19.124031	3.676962	1.594	−0.0644
21	19.636256	3.690616	1.573	−0.0023

**Table 7:** Preferred combination of process parameters.

Parameter	*T*_h_/h	*T*_k_/h	*T*_c_/h	Time-I/h	Time-II/h
Value	6	2	3	11.00	15.85

**Table 8:** Autoclave parameters for experiments.

Parameter	Value
Pattern	SEET-R024
Norm/mm	Φ2200×6000
*P*_max_/MPa	0.9
*T*_max_/K	524.15
Work media	Compressed air
Lifting/buck rate MPa/min	0.05/0.1
Heating/cool rate K/min	277.150
Air pressure/MPa	6.000
Vacuum/MPa	−0.098
Temperature uniformity/K	±277.150

**Table 9:** Stacking sequences of plies in walled panel.

Region	Skin Area 1	Skin Area 2	Beam
Stiffened	[(45/0/−45)_2_/(45/0/−45/0)_2_]	[(45/−45/0/45/−45/0)_2_/(45/0/−45/0/45/0/−45/0)_S_]	[(45/0/−45/0/45/0/−45/90)_S_]_2_
Transition	[45/0/−45/(45/0/−45/0)_2_]	[45/−45/0/45/−45/0/(45/0/−45/0/45/0/−45)_S_]	[45/0/−45/0/45/0/−45/90]_SS_
Thin-walled	[45/0/−45/0]_2_	[(45/0/−45/0/45/0/−45/90)_S_/(45/0/−45/0)_2_]	[(45/0_2_/−45/0/45/0/−45/90)_S_/45/−45/0/45/−45/0]

**Table 10:** The measured and simulation value of samples.

ID	Simulation/mm	Experiential/mm	Error/%
A	3.200	3.181	0.59%
B		3.185	0.47%
C		3.184	0.50%
D		3.183	0.53%
E	0.059	0.060	1.7%
F		0.062	5.1%
G		0.058	1.7%
H		0.059	0%

**Table 11:** Comparison of the deviation amount of the numerical simulation at the selection point of the wall plate member and the experimental results.

Point	A001	A002	A003	A004	A005
Experimental/mm	1.711	1.113	0.755	−1.008	−1.506
Simulation/mm	2.021	1.229	0.899	−0.844	−1.282
Error/%	18.13	10.44	19.17	16.34	14.89

## Data Availability

The raw data supporting the conclusions of this article will be made available by the authors on request due to privacy.
